# Determination of the median effective dose (ED₅₀) of oliceridine combined with propofol for inhibiting gastroscope insertion responses in adult patients undergoing painless gastroscopy

**DOI:** 10.3389/fmed.2026.1877936

**Published:** 2026-08-05

**Authors:** Xiaojing Zhang, Weimin Qin, Xinying Zhang, Wenbo Liang, Zhiqiang Ren, Qing Ma, Li Zhou, Longbin Zheng

**Affiliations:** 1 Department of Anesthesiology, Sir Run Run Hospital, Nanjing Medical University, Nanjing, Jiangsu, China; 2 Department of Anesthesiology, The People’s Hospital of Rugao, Rugao Hospital Affiliated to Nantong University, Rugao, Jiangsu, China; 3 Department of Anesthesiology, Affiliated Hospital of Jiangsu University, Zhenjiang, Jiangsu, China

**Keywords:** anesthetic efficacy, median effective dose, oliceridine, painless gastroscopy, propofol

## Abstract

**Background:**

Propofol is the most frequently administered intravenous anesthetic in painless gastroscopy. Oliceridine, a novel *μ*-opioid receptor agonist, exhibits analgesic effects while reducing adverse reactions. The combination of propofol and oliceridine has shown promising efficacy in clinical practice. However, the exploration of the specific effective dosage of oliceridine in painless gastroscopy is still in its early stages. This study aimed to determine the median effective dose (ED₅₀) of oliceridine when combined with propofol during painless gastroscopy.

**Methods:**

Patients aged 18–85 years with American Society of Anesthesiologists (ASA) physical status I to III undergoing painless gastroscopy were enrolled. They received an intravenous injection of oliceridine, followed by propofol at a dose of 1.5 mg/kg. The initial dose of oliceridine for the first patient was 20 μg/kg, and subsequent doses were adjusted using Dixon’s up-and-down method based on the previous patient’s response to gastroscope insertion—with a 1 μg/kg increase or decrease depending on the observed response. Data collection continued until 7 crossover points were obtained. Probit regression and bootstrapping methods were used to calculate the ED₅₀ and its 95% confidence intervals (CI). Hemodynamic parameters and perioperative adverse events were recorded.

**Results:**

A total of 24 patients were included in the study. The ED₅₀ of oliceridine combined with propofol (1.5 mg/kg) for inhibiting the response to gastroscope insertion in patients undergoing painless gastroscopy was 19.33 μg/kg (95% CI, 17.41 ~ 21.98 μg/kg). The vital signs of all patients remained within the predefined acceptable limits, and no serious adverse events occurred.

**Conclusion:**

The ED₅₀ of oliceridine combined with propofol (1.5 mg/kg) for inhibiting gastroscope insertion responses in adult patients undergoing painless gastroscopy is 19.33 μg/kg.

**Clinical trials registration:**

ChiCTR2500105523.

## Introduction

In recent years, painless gastroscopy has become the most important diagnostic and therapeutic method for digestive tract diseases due to its advantages such as alleviating patient suffering and improving examination compliance (1). The smooth implementation of painless gastroscopy is closely related to the safety of anesthesia protocols (2). Therefore, it is necessary to reduce the risk of respiratory and circulatory depression during anesthesia management for painless gastroscopy and shorten the postoperative awakening time.

As the most commonly used intravenous anesthetic in painless gastroscopy, propofol has numerous advantages including rapid onset, quick awakening, and a high metabolic clearance rate (3). However, the analgesic effect of propofol is weak (4). To achieve an ideal anesthetic effect, the combination of propofol and opioids is the most frequently used anesthesia regimen (5). The use of opioids can effectively reduce the dosage of propofol, which not only enhances the anesthetic effect but also mitigates the safety risks associated with high doses of propofol (6).

Oliceridine is a novel *μ*-opioid receptor agonist with selective activation of G protein and *β*-arrestin signaling pathways (7). Studies have reported that oliceridine not only provides excellent analgesic effects but also reduces the incidence of adverse events such as respiratory depression, nausea, and vomiting compared to sufentanil (8). Recently, the safety and efficacy of oliceridine combined with propofol in painless endoscopy have been confirmed (9). However, the exploration of the specific effective dosage of oliceridine in painless gastroscopy is still in its infancy. When used in combination with propofol for painless gastroscopy, the ED₅₀ of oliceridine that can effectively inhibit intraoperative body movement responses has not yet been clarified.

The determination of the median effective dose is an important prerequisite for individualized medication research. Therefore, this study intends to adopt the adopt the Dixon sequential up-and-down method to observe intraoperative gastroscopic insertion responses in patients undergoing painless gastroscopy with different doses of oliceridine combined with propofol, calculate the ED₅₀ of oliceridine and its 95% CI, and provide an objective basis for clinical practice.

## Methods

### Ethics and trial registration

This prospective, single-center study was approved by the Medical Ethics Committee of Sir Run Run Hospital, Nanjing Medical University (2025-SR-017), and registered with the Chinese Clinical Trial Registry (ChiCTR2500105523, 04/07/2025). A designated anesthesiologist screened and enrolled eligible patients, and written informed consent was obtained from patients or their family members. The study protocol was conducted in accordance with the Declaration of Helsinki guidelines and the International Conference on Harmonization guidelines for Good Clinical Practice.

### Inclusion and exclusion criteria

All patients undergoing painless gastroscopy from August 11, 2025, to December 15, 2025, were screened for enrollment. Patients were included if they met the following criteria: age 18–85 years and ASA classification I to III. The exclusion criteria were as follows: (1) History of allergy to any of the study medications; (2) Contraindications to general anesthesia; (3) Presence of heart, lung, liver, kidney, blood, or other major systemic diseases that may affect pharmacokinetics; (4) Communication or cognitive impairment.

### Anesthesia protocol

All patients fasted routinely before painless gastroscopy and received no preoperative medication. After entering the operating room, peripheral intravenous access was established immediately, and patients were placed in the left lateral position. Noninvasive blood pressure (NIBP), electrocardiogram (ECG), heart rate (HR), and pulse oxygen saturation (SpO₂) were monitored routinely. Oxygen was administered at 2–3 L/min via a nasal catheter, and end-expiratory carbon dioxide (PETCO₂) was monitored continuously. Rescue medication and emergency airway equipment were prepared in advance.

For anesthesia induction, oliceridine was initially administered as a single intravenous bolus at a dosage of 20 μg/kg. Two minutes later, propofol was injected intravenously at a dosage of 1.5 mg/kg. The endoscopist began the procedure when the patient’s eyelash reflex disappeared and the Modified Observer’s Assessment of Alertness/Sedation (MOAA/S) score was ≤ 1. All anesthetic procedures were performed by the same experienced anesthesiologist, and all surgical procedures were performed by the same experienced endoscopist.

During the operation, if SpO₂ fell below 90% or PETCO₂ exceeded 50 mmHg for more than 10 s, airway-opening maneuvers such as chin lift, head-tilt, and jaw-thrust were performed immediately. If hypoxemia persisted despite these interventions, the operation was stopped immediately, the gastroscope was removed, and pure oxygen was administered via a mask (with positive pressure ventilation if necessary). Ephedrine 6 mg was injected when systolic blood pressure (SBP) was below 30% of the baseline level, and atropine 0.5 mg was injected when HR was below 45 beats/min. Other perioperative adverse events were recorded and managed in accordance with clinical operation standards.

Following the completion of painless gastroscopy, patients were transferred to the post-anesthesia care unit (PACU) for an observation period of no less than 30 min. Patients were permitted to leave the operating room when they achieved full awakening, a Post-Anesthetic Discharge Scoring System (PADSS) score of ≥9, and stable vital signs.

### Determination of the ED₅₀ for oliceridine

The median effective dose of oliceridine combined with propofol was determined using the Dixon sequential method. Based on preliminary experimental results and relevant studies, the initial dosage of oliceridine was 20 μg/kg. If a positive response (defined as swallowing, coughing, retching, or body movement) occurred during gastroscope insertion in the previous patient, the dosage of oliceridine for the next patient was increased by 1 μg/kg. If a negative response (absence of the above reactions) was observed, the dosage was decreased by 1 μg/kg. If a patient exhibited a positive response, an additional 0.5 mg/kg of propofol was administered. The trial was terminated when 7 inflection points were formed in the alternating wave of positive and negative responses to gastroscope insertion.

### Outcomes

HR, mean arterial pressure (MAP), SpO₂, and respiratory rate (RR) were monitored at the following time points: before anesthesia (T0), before gastroscope insertion (T1), when the gastroscope passed through the pharynx (T2), when the gastroscope reached the descending part of the duodenum (T3), and when the gastroscope was withdrawn from the pharyngeal cavity (T4). Intraoperative adverse events recorded included hypotension (SBP decreased by more than 20% from baseline or < 90 mmHg), bradycardia, subclinical respiratory depression (90% ≤ SpO₂ ≤ 95%), hypoxia (75% < SpO₂ < 90% for < 60 s), and severe hypoxia (SpO₂ < 75%, or 75% < SpO₂ < 90% for > 60 s). Post-anesthesia adverse events recorded included headache, dizziness, nausea, and vomiting within 2 h after the operation.

### Statistical analysis

Statistical analysis was performed using SPSS 23.0 software. The Shapiro–Wilk test was used to assess the normality of data distribution. Normally distributed continuous data were expressed as mean ± standard deviation (x ± s). Non-normally distributed continuous variables were presented as median and interquartile range, and categorical variables were presented as n (%). Data were compared between groups using unpaired t-tests, chi-squared tests, Fisher’s exact test, or Mann–Whitney U-test, as appropriate. The ED₅₀ and its 95% CI were calculated using the Dixon sequential trial formula. Sequential line graphs and dose–response curves were plotted using GraphPad Prism 10 software. *p* < 0.05 was considered statistically significant.

## Results

**Figure 1:**
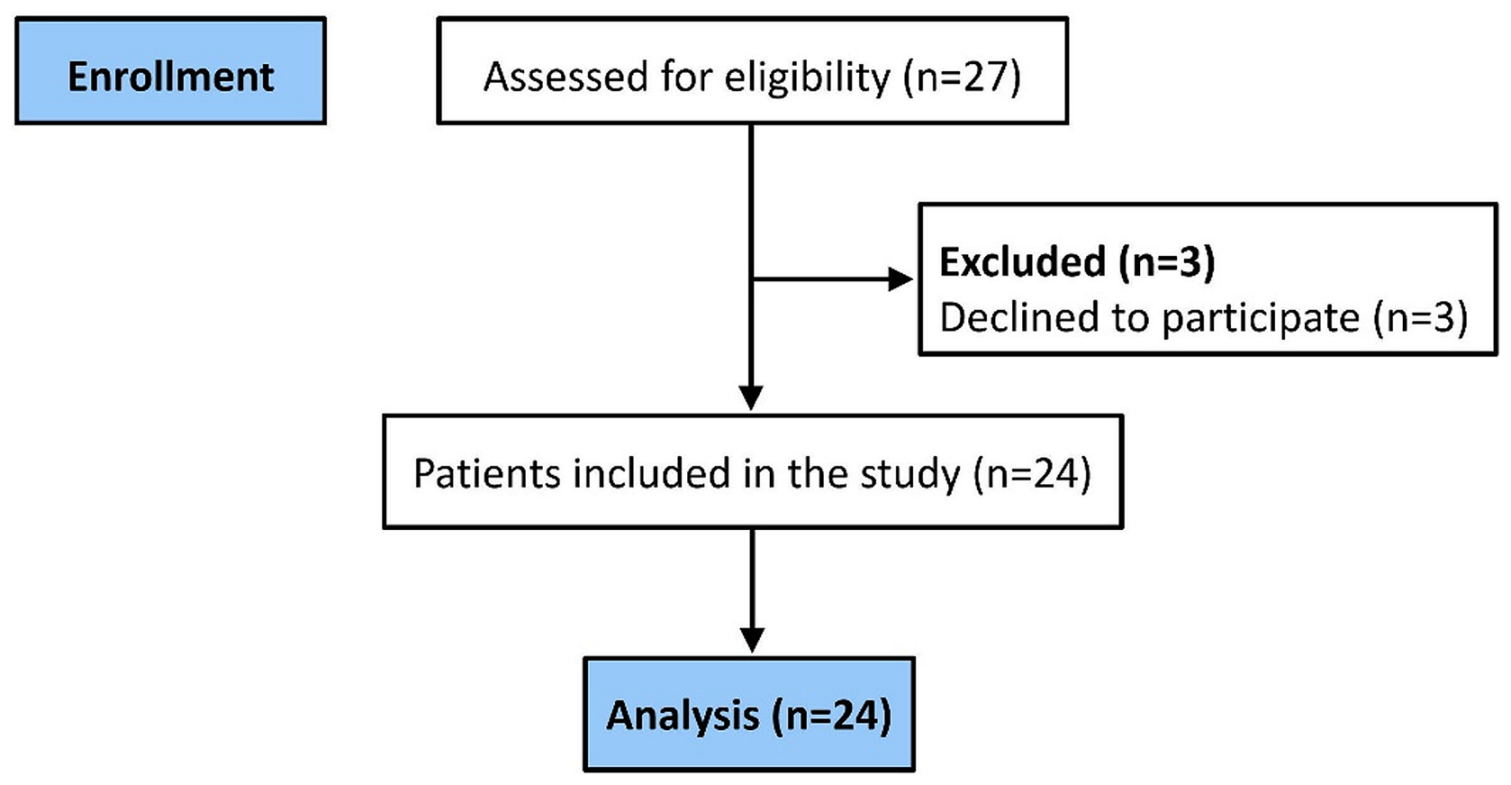
Study population flow diagram.

From August 11, 2025, to December 15, 2025, a total of 27 patients met the inclusion criteria. Of these, 3 patients withdrew consent, resulting in 24 patients being enrolled in the study (Figure 1). The demographic data and clinical characteristics of the patients are presented in Table 1.

**Table 1:** Demographic data and anesthesia/surgery-related indicators of patients.

Characteristics	Values
Gender (Male/Female)	15 ∕ 9
Age (years)	38.8 ± 7.8
BMI (kg/m^2^)	22.1 ± 2.2
ASA classification (I ∕ II ∕ III)	6 ∕ 18 ∕ 0
Total propofol dose (mg)	137.7 ± 7.4
Gastroscopy time (min)	6.1 ± 1.0
Anesthesia recovery time (min)	1.7 ± 0.6

Data are presented as mean±standard deviation, or number.

BMI, Body mass index; ASA, American Society of Anesthesiologists.

**Figure 2:**
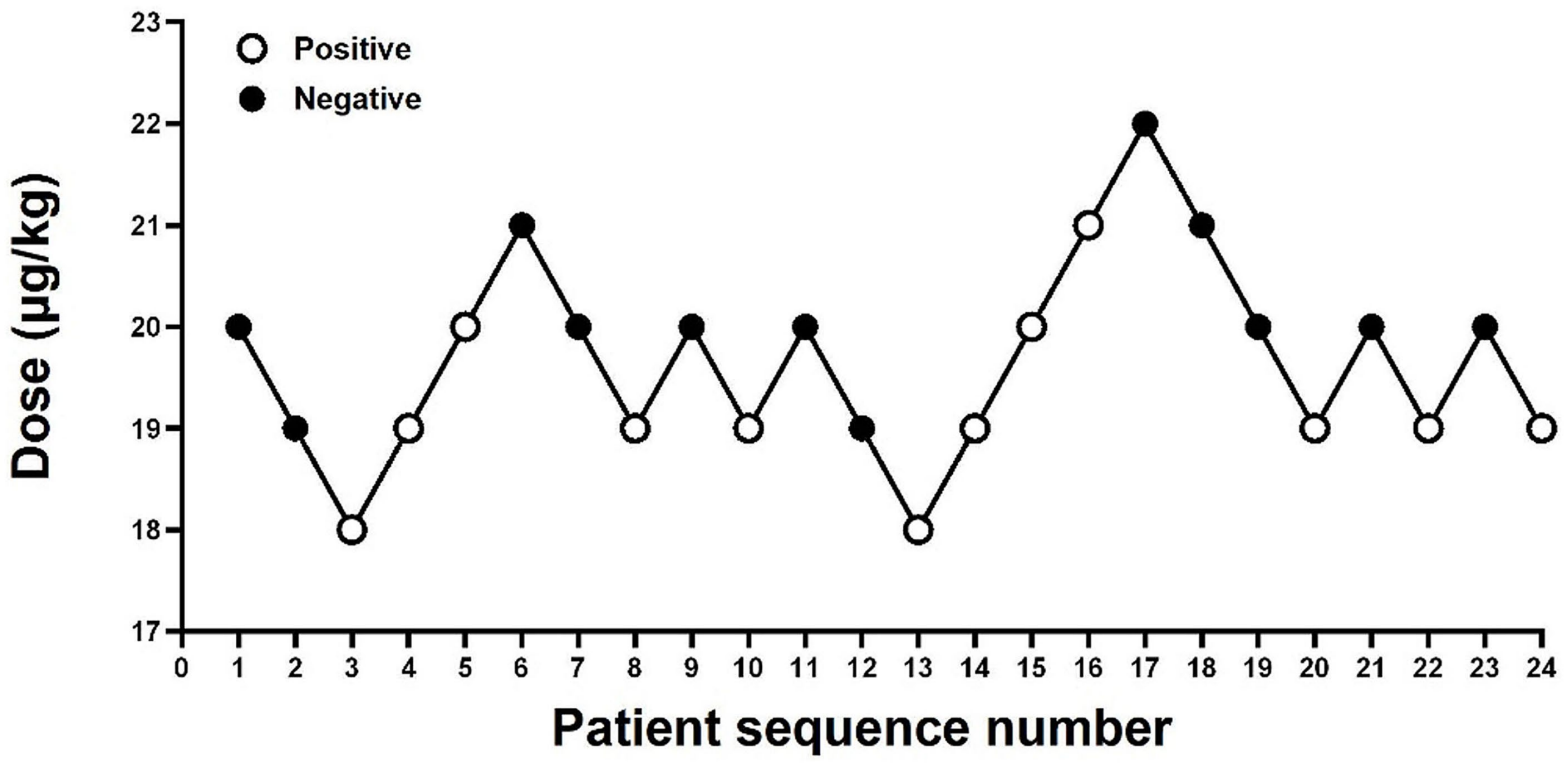
Dixon and Massey up-and-down plot line chart. The sequence of patients receiving oliceridine.

**Figure 3:**
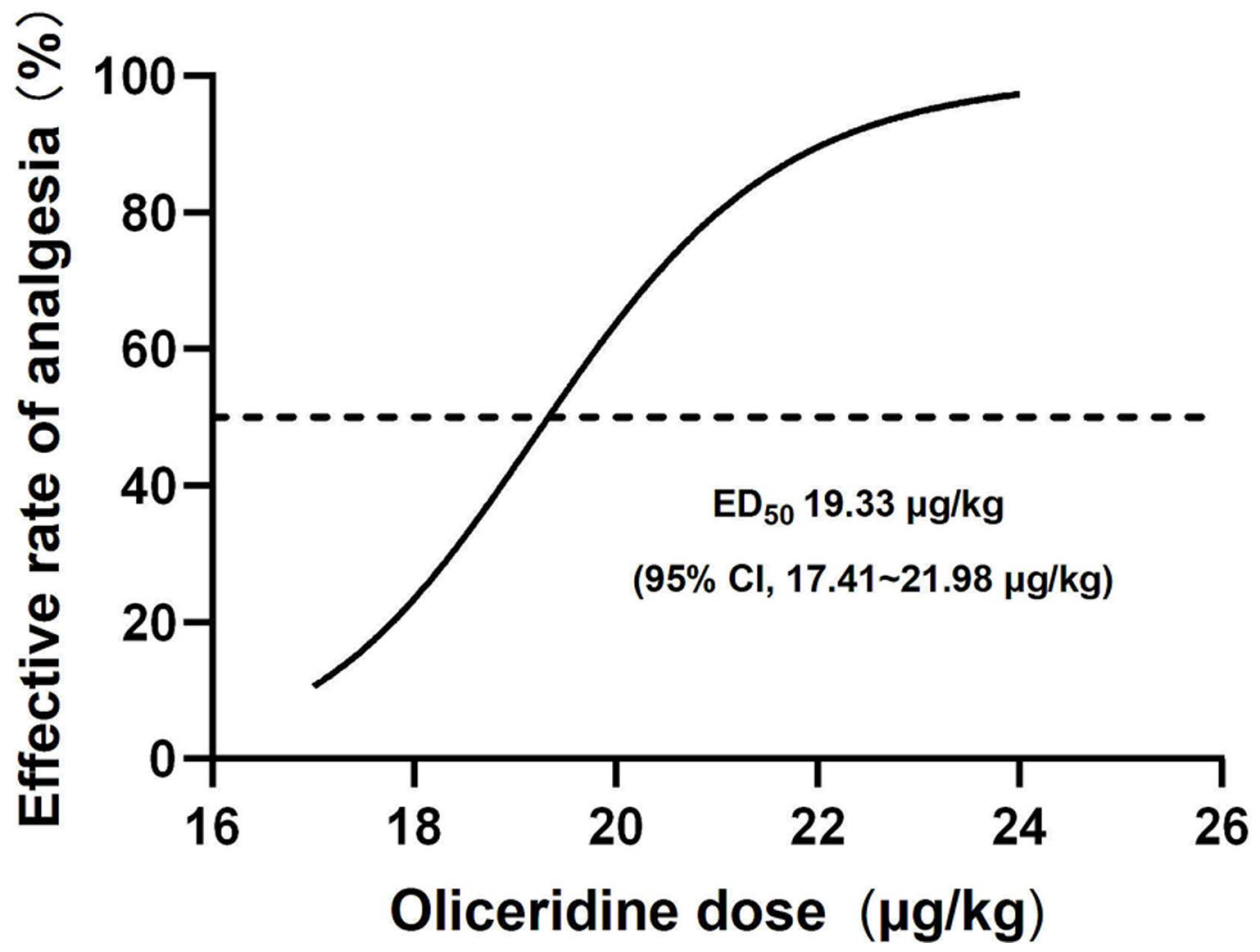
Dose–response curve of oliceridine for inhibiting gastroscopic insertion responses in patients undergoing painless gastroscopy.

The sequential doses of oliceridine combined with propofol using the up-and-down method are illustrated in Figure 2. Probit regression analysis showed that the ED₅₀ of oliceridine combined with propofol during painless gastroscopy was 19.33 μg/kg (95% CI, 17.41 ~ 21.98 μg/kg). The dose–response curve of oliceridine is shown in Figure 3.

MAP and HR at T1 were significantly lower than those at T0 (*p* < 0.05), but there were no significant differences in MAP and HR at T2, T3, and T4 compared to T0. There were no significant differences in SpO₂ and RR at T1, T2, T3, and T4 compared to T0 (Table 2). Among the 24 patients, 2 had bradycardia (Table 3). No other adverse reactions such as hypoxemia, dizziness, nausea, or vomiting occurred (Table 4), and no patient required antagonist rescue.

**Table 2:** Comparison of HR, MAP, SpO_2_, and RR at different time points in patients undergoing painless gastroscopy.

Indicator	T0	T1	T2	T3	T4	*p* value
MAP	88.6 ± 6.7	82.0 ± 5.2^a^	87.6 ± 6.4	84.8 ± 8.6	88.2 ± 5.3	0.0022
HR	71.0 ± 11.3	62.8 ± 9.0^a^	74.3 ± 9.5	71.1 ± 11.7	71.5 ± 9.1	0.0025
SpO_2_	99.4 ± 0.8	99.0 ± 1.0	98.1 ± 1.1	99.2 ± 0.7	99.3 ± 0.8	0.7055
RR	14.5 ± 1.5	13.7 ± 1.4	14.0 ± 1.2	14.4 ± 1.3	14.6 ± 1.3	0.0639

Data are presented as mean ± standard deviation. Compared with T0, ^a^*p* < 0.05.

**Table 3:** Incidence of adverse reactions during anesthesia.

Hypotension	Bradycardia	Subclinical respiratory depression	Hypoxia	Severe hypoxia
0	2	0	0	0

**Table 4:** Incidence of postoperative adverse reactions.

Headache	Dizziness	Nausea	Vomiting
0	0	0	0

## Discussion

Determining the optimal dosage of anesthetics is crucial for anesthesia management during painless gastroscopy. ED₅₀ is the most commonly used parameter for exploring the dose-effect relationship of drugs and is routinely applied in clinical trials (10). This study used the Dixon sequential method combined with probit regression analysis to confirm that the ED₅₀ of oliceridine combined with propofol for inhibiting gastroscope insertion responses during painless gastroscopy is 19.33 μg/kg (95% CI, 17.41 ~ 21.98 μg/kg). Meanwhile, the hemodynamic effects and adverse reactions were systematically evaluated, providing precise dosage references and safety evidence for clinical anesthetic practice.

Propofol is the most commonly used anesthetic in general anesthesia, with a recommended loading dosage of 1.5–2.5 mg/kg for adult patients (2). When administered in combination with opioids, the suggested loading dosage of propofol is 1.5 mg/kg (3). Therefore, 1.5 mg/kg of propofol combined with oliceridine was selected for anesthesia induction in this study. Based on pre-experimental data and relevant references, the initial dosage of oliceridine was set at 20 *μ*g/kg with an adjacent dose difference of 1 μg/kg. Studies have reported that the onset time of oliceridine is 1–3 min, and it reaches peak effect within 5–15 min (11). According to its onset time and peak time, administering propofol 2 min after oliceridine injection is reasonable, as it exhibits a promising synergistic effect in inhibiting the gastroscope insertion response.

As a novel μ-opioid receptor agonist, oliceridine has a unique pharmacological mechanism that provides analgesic effects while reducing the adverse reactions of traditional opioids (5). Thus, oliceridine has been widely used in general anesthesia for minor surgeries (6). Studies have reported that oliceridine has a significantly lower incidence of respiratory depression compared to traditional opioids, which is consistent with the findings of the present study (7). In this study, none of the patients experienced respiratory depression. This may be mainly attributed to oliceridine being a biased *μ*-opioid receptor agonist, which exhibits higher selectivity for specific subtypes or signaling pathways of the μ-opioid receptor with minimal activation of the *β*-arrestin pathway.

Hemodynamic instability is the most common intraoperative adverse event during painless gastroscopy (12). In this study, hemodynamic monitoring showed that both MAP and HR at T1 were significantly lower than those at T0. However, there were no significant differences in MAP and HR at T2, T3, and T4 compared to T0. These results are consistent with the literature on general anesthesia induction with propofol. The decreases in MAP and HR are mainly associated with the vasodilatory effect of propofol. Nevertheless, the fluctuation range was within the clinically safe range; no severe hypotension was observed, and bradycardia occurred in only 2 patients, which did not require clinical intervention. This may be attributed to oliceridine having a milder impact on the cardiovascular system compared to traditional opioids. Furthermore, SpO₂ and RR showed no statistically significant changes throughout the procedure, indicating that the medication regimen has no adverse effects on respiratory function. Therefore, the data confirm the safety and feasibility of combining propofol and oliceridine for non-intubated general anesthesia patients.

Traditional opioids such as morphine and fentanyl are often associated with a high incidence of nausea and vomiting in general anesthesia for painless digestive endoscopy (13). Studies have reported that oliceridine reduces *β*-arrestin-mediated adverse reactions by selectively activating the G protein signaling pathway of *μ*-opioid receptors, thereby lowering the risk of gastrointestinal reactions (14). Consistent with this, no adverse reactions such as dizziness, nausea, or vomiting occurred among the 24 patients in this study. This advantage is of great significance for improving postoperative patient comfort and shortening recovery time, and it is particularly aligned with the application requirements of the Enhanced Recovery After Surgery (ERAS) concept in painless digestive endoscopy examinations.

This study has certain limitations. Firstly, the small sample size and short study period may limit the external validity of the results. Future prospective, multi-center studies with large samples are needed to verify the stability of the ED₅₀ of oliceridine and its long-term safety. Secondly, this study excluded patients with heart, lung, liver, kidney, blood, or other major systemic diseases. Subsequent studies should expand the study population to clarify dosage adjustment schemes for different groups, thereby providing a more accurate medication basis for clinical practice.

## Conclusion

In conclusion, the ED₅₀ of oliceridine combined with 1.5 mg/kg propofol for inhibiting gastroscopic insertion responses in adult patients undergoing painless gastroscopy is 19.33 μg/kg. Additionally, this combination is confirmed to be a safe and effective anesthesia regimen worthy of further clinical application.

## Data Availability

The original contributions presented in the study are included in the article/supplementary material, further inquiries can be directed to the corresponding author.
